# Development of Accessory Cells in B-Cell Compartments Is Retarted in B-Cell-Depleted Fetal Sheep

**DOI:** 10.1155/1998/27025

**Published:** 1998

**Authors:** Charles Mcl. Press, John D. Reynolds, Susan J. McClure, Thor Landsverk

**Affiliations:** 1 Department of Morphology, Genetics and Aquatic Biology Norwegian College of Veterinary Medicine P.O. Box 8146, Dept. 0033 Oslo Norway; 2 Department of Medical Physiology University of Calgary 3330 Hospital Dr. NW Alberta Calgary T2N 4N1 Canada; 3 CSIRO McMaster Laboratory Division of Animal Production Locked Bag 1 Delivery Centre Blacktown NSW 2148 Australia

**Keywords:** Fetal sheep, accessory cells, follicular dendritic cells, macrophages, B cells, immunohistochemistry, enzyme histochemistry

## Abstract

Accessory-cell populations in the lymphoid tissues of fetal sheep were investigated following depletion of B cells. An intraperitoneal injection of an anti-IgM antibody early in gestation resulted in a marked depletion of IgM^+^ cells in lymphoid tissues. Immune and enzyme histochemical techniques were used to identify accessory-cell populations in the ileal Peyer's patch, spleen, and lymph nodes of B-cell-depleted fetal sheep. The rudimentary follicles in the ileal Peyer's patch showed strong enzyme reactivity for 5′ nucleotidase, indicating the presence of follicular dendritic cells (FDCs). Enzyme reactivities for FDCs in primary follicles of the spleen and lymph nodes were absent, as were reactivities for metallophilic macrophages in the marginal zone of the spleen. MgATPase reactivity associated with dendritic-cell populations in the gut-associated lymphoid tissues was detected. A monoclonal antibody against complement receptor-2 (CD21) reacted with FDCs in the rudimentary follicles of the ileal Peyer's patch and immature FDCs in lymph nodes. The results suggest that the development of accessory-cell populations in B-cell compartments of peripheral but not central lymphoid tissues is dependent on the presence of B cells.


*Developmental Immunology*, 1998, Vol. 6, pp. 223–231
Reprints available directly from the publisher
Photocopying permitted by license only

© 1998 OPA (Overseas Publishers Association)
N.V. Published by license under the
Harwood Academic Publishers imprint,
part of the Gordon and Breach Publishing Group
Printed in Malaysia

### Development of Accessory Cells in B-Cell Compartments Is Retarded in B-Cell-Depleted Fetal Sheep

CHARLES McL. PRESS[a*], JOHN D. REYNOLDS[b], SUSAN J. McCLURE[c] and THOR LANDSVERK[a]

[a]*Department of Morphology, Genetics and Aquatic Biology, Norwegian College of Veterinary Medicine, P.O. Box 8146, Dept. 0033, Oslo, Norway*; [b]*Department of Medical Physiology, University of Calgary, 3330 Hospital Dr. NW, Calgary, Alberta, Canada T2N 4N1*; [c]*CSIRO McMaster Laboratory, Division of Animal Production, Locked Bag 1, Delivery Centre, Blacktown, NSW, 2148, Australia*

*(Received 6 August 1996; Accepted 18 August 1997; In final form 20 September 1997)*

Accessory-cell populations in the lymphoid tissues of fetal sheep were investigated following depletion of B cells. An intraperitoneal injection of an anti-IgM antibody early in gestation resulted in a marked depletion of $IgM^+$ cells in lymphoid tissues. Immune and enzyme histochemical techniques were used to identify accessory-cell populations in the ileal Peyer's patch, spleen, and lymph nodes of B-cell-depleted fetal sheep. The rudimentary follicles in the ileal Peyer's patch showed strong enzyme reactivity for 5′nucleotidase, indicating the presence of follicular dendritic cells (FDCs). Enzyme reactivities for FDCs in primary follicles of the spleen and lymph nodes were absent, as were reactivities for metallophilic macrophages in the marginal zone of the spleen. MgATPase reactivity associated with dendritic-cell populations in the gut-associated lymphoid tissues was detected. A monoclonal antibody against complement receptor-2 (CD21) reacted with FDCs in the rudimentary follicles of the ileal Peyer's patch and immature FDCs in lymph nodes. The results suggest that the development of accessory-cell populations in B-cell compartments of peripheral but not central lymphoid tissues is dependent on the presence of B cells.

*Keywords:* Fetal sheep, accessory cells, follicular dendritic cells, macrophages, B cells, immunohistochemistry, enzyme histochemistry

### INTRODUCTION

The role of accessory cells such as follicular dendritic cells, macrophages, and dendritic cells in promoting and regulating immune responses is well-known. However, the ontogeny of accessory cells and their developmental relationship with other cells that populate lymphoid tissues is less understood (O'Neill, 1994). The investigation of the emergence and subsequent development of follicular dendritic cells (FDCs) in the primary follicles of fetal sheep lymph nodes showed that prior to birth, FDCs were ultrastructurally immature and that final differentiation to a mature FDC was coincident with the postnatal germinal center reaction (Halleraker et al., 1994). Although the final differentiation to a mature phenotype occurred in early postnatal life, the closer study of patterns of enzyme reactivity and the morphometric analysis of the size of lymph node primary follicles showed that FDCs underwent modification during the

*Corresponding author.

last third of gestation and it was concluded that stromal cells in primary follicles of fetal lymph nodes were continuously developing structures (Halleraker et al., 1994). As sheep are only exposed to exogenous nonself antigens after birth because of the protection provided by a syndesmochorial placenta (Brambell, 1970; Boyd et al., 1976), it follows that factors other than exogenous nonself antigens are responsible for this modification.

The interdependence of lymphoid-cell differentiation and stromal-cell development has been the subject of a number of studies. In man, rat and sheep, the early occurrence of FDCs was concomitant with B-cell aggregation and the appearance of primary follicles (Markgraf et al., 1982; Kroese et al., 1987; Halleraker et al., 1994). Indeed, Kroese et al. (1987) were drawn to conclude that in the rat, FDCs developed independently of the presence of antigen and of follicular type B cells and that mature FDCs created at certain locations a microenvironment conducive to primary follicle formation. However, following the study of lymph nodes in B-cell-depleted mice, Cerny et al. (1988) reported a different relationship in that mature B cells were found to be required for the development of mature FDCs.

The recent demonstration that fetal sheep can be depleted of B cells following anti-IgM treatment early in ontogeny (Press et al., 1996) has allowed the investigation of the development of lymphoid and accessory-cell populations in a species shielded from exogenous antigen during gestation. In addition to FDCs in primary follicles, other accessory-cell populations that share a close association with B cells include the FDCs of gut-associated lymphoid follicles and macrophages of the marginal zone of the spleen. The aim of the present study was to characterize, using a panel of enzyme and immunohistochemical techniques, the accessory-cell populations in the lymphoid tissues of B-cell-depleted fetal sheep.

### RESULTS

### Ileal Peyer's Patch Follicles

The small follicles in the ileal Peyer's patch of the B-cell-depleted fetuses demonstrated intense reactivity for the enzyme 5′nucleotidase (5′N), which was similar to the intensity of reactivity shown in the ileal Peyer's patch follicles of the untreated litter mates and the age-matched control fetuses (Figure 1). There was intense reactivity for magnesium-dependent adenosine triphosphatase (MgATPase) in some cells of the dome and interfollicular areas in the ileal Peyer's patch of the B-cell-depleted fetuses (Figure 1). The dome region of ileal Peyer's patch of the B-cell-depleted fetuses showed increased reactivity for acid phosphatase whereas there was an absence of reactivity in the rudimentary follicle (Figure 1) There was also some reactivity for nonspecific esterase in the dome and interfollicular areas (not shown).

A double immunofluorescence technique showed that the rudimentary follicles of the ileal Peyer's patch in the B-cell-depleted fetuses showed strong staining for complement receptor-2 (CD21) and did not stain for IgM (not shown).

### Primary Follicles in the Spleen and Lymph Nodes

The spleen and lymph nodes of the B-cell-depleted fetuses did not show the characteristic patterns of enzyme reactivity for 5′N, MgATPase, nonspecific esterase, and alkaline phosphatase that are associated with primary follicles (Figure 1). Serial step sectioning of the spleen and lymph nodes combined with staining for 5′N reactivity did not identify primary follicles in the B-cell-depleted fetuses. Primary follicles were readily identifiable by their typical pattern of enzyme reactivities in the control fetuses (Figure 1).

The spleen and lymph nodes of B-cell-depleted fetuses contained a small population of $CD21^+$ cells. In the lymph nodes, most of these cells were scattered in the cortex, but some cells tended to form small accumulations. Double immunofluorescence showed that the accumulations consisted mainly of $CD21^+$, weakly $IgM^+$ cells, which presumably represented B cells. However, some $CD21^+$ cells in these accumulations did not stain for IgM and may represent precursor FDCs (Figure 2).

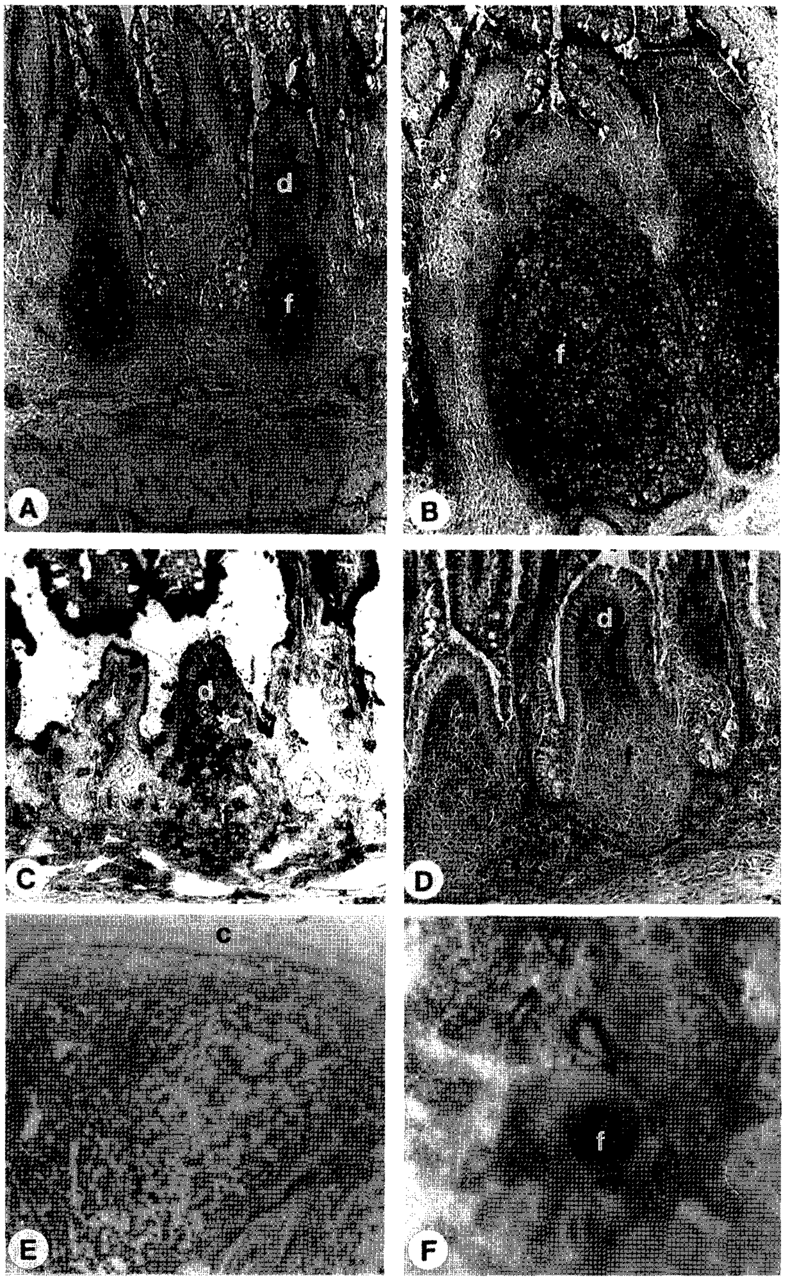

FIGURE 1 (A) Ileal Peyer's patch of a B-cell-depleted fetal lamb showing intense enzyme reactivity in the rudimentary follicles (f). d = dome. 5′N. ×120. (B) Ileal Peyer's patch of a control lamb showing strong enzyme reactivity in the large follicles (f). d = dome. 5′N ×120. (C) Ileal Peyer's patch of a B-cell-depleted fetal lamb showing strong enzyme reactivity in cells in the dome (d) of a rudimentary follicle (f). i = interfollicular area. MgATPase. ×120. (D) Ileal Peyer's patch of a B-cell-depleted fetal lamb showing strong enzyme reactivity in the dome (d) and little to no reactivity in the rudimentary follicle (f). i = interfollicular area. ACP. ×120. (E) Distal jejunal lymph node of a B-cell-depleted fetal lamb showing an absence of the intense enzyme reactivity associated with primary follicles. c = capsule. 5′N. ×95. (F) Distal jejunal lymph of a control lamb showing the intense enzyme reactivity in a primary follicle (f). 5′N. ×95.

### Marginal Zone of the Spleen

Strong cellular reactivity for nonspecific esterase and acid phosphatase associated with metallophilic macrophages that was present in the marginal zone of the control fetuses was absent in the B-cell-depleted fetuses (Figure 3). A cell population showing weaker reactivity for nonspecific esterase and acid phosphatase was present in the marginal zone. Cell in the red pulp of the spleen showed less reactivity for acid phosphatase (Figure 3). Reactivity for alkaline phosphatase in the marginal sinus endothelium was present in both the B-cell-depleted and control fetuses (Figure 3).

### DISCUSSION

The present study shows that accessory-cell populations in B-cell compartments of anti-IgM-treated fetal sheep were retarded in development. However, differences were observed between stromal-cell populations in the spleen and lymph nodes and stromal cells in the ileal Peyer's patch. A previous study has shown that the ileal Peyer's patch of B-cell-depleted fetal sheep did not contain B cells and only a small, scattered population of B cells was present in the spleen and lymph nodes (Press et al., 1996). With the depletion of B cells, conventional secondary lymphoid tissues of the spleen and lymph nodes did not show typical patterns of enzyme reactivity for FDCs in primary follicles, whereas the FDCs in the ileal Peyer's patch continued to express strong reactivity for 5′N (Halleraker et al., 1990; 1994; Nicander et al., 1991). In sheep, the ileal Peyer's patch functions as an organ of post-rearrangement diversification (Reynaud et al., 1995; 1991; Weill and Reynaud, 1996) and is responsible for the vast majority of B-cell production (Reynolds and Morris, 1983; Gerber et al., 1986). The growth and function of the ileal Peyer's patch has been related to processes of epithelial differentiation (Landsverk, 1987; Landsverk et al., 1987, 1990). In contrast to ileal Peyer's patch follicles, 5′N reactivity in the primary follicles of the spleen and lymph nodes was not detected in B-cell-depleted fetuses. Halleraker et al. (1990) observed similarities between the enzyme reactions of stromal cells at early stages of primary follicle development and those in Peyer's patch follicles of fetal and neonatal lambs. However, the present observations would argue for a difference in the development and differentiation of these two stromal-cell populations and be consistent with the observed differences between Peyer's patches and germinal centers (Reynolds, 1985).

The primary follicles of the spleen and lymph-node cortex are accumulations of B cells that include small and medium lymphocytes, lymphoblastlike cells, macrophages and FDCs (Nossal et al., 1968; Sakuma et al., 1981; Weiss, 1988; Halleraker et al., 1994) and that after antigenic stimulation are the site of the germinal center reaction. Kroese et al. (1987) postulated that the development of FDCs in rats was independent of the presence of antigen and (follicular) B cells and suggested that mature FDCs created a microenvironment conducive to primary follicle formation and the subsequent generation of a germinal center. However, the findings in anti-IgM-treated rats (Kumararatne et al., 1981), mice (Cerny et al., 1988) and now fetal sheep argue against this suggestion and instead demonstrate that in the absence of B cells primary follicles and their population of FDCs fail to develop. Whereas precursor FDCs were present in B-cell-depleted mice, the transition of a precursor FDC to a FDC was associated with the presence of mature B cells (Cerny et al., 1988). The present demonstration of $CD21^+$, IgM-negative cells among small accumulations of B cells in the lymph-node cortex also suggests the presence of precursor FDCs in B-cell-depleted fetal sheep, although ultrastructural and reconstitution experiments will be needed to confirm whether these cells represent precursor FDCs.

The $MgATPase^+$ dendritic cells are the major accessory cell population in the T-cell compartments of gut-associated lymphoid tissue (Halleraker et al., 1990), and as found in anti-IgM treated mice (Cerny et al., 1988), dendritic-cell populations appeared to be unaffected by B-cell depletion. Dendritic cells derive from bone marrow stem cells (Steinman et al., 1975; Reid et al., 1992) and are closely related to the development of T-cell populations (O'Neill, 1994).

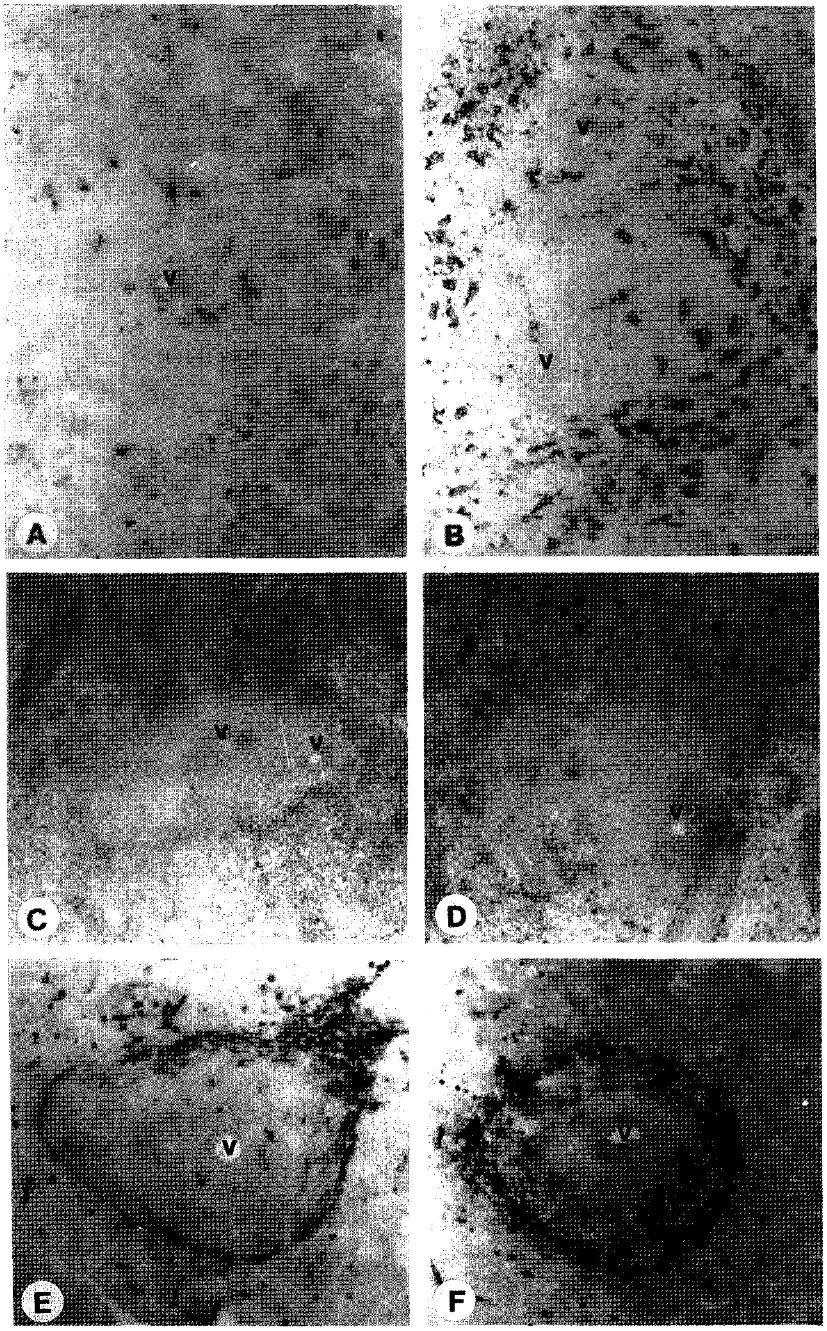

FIGURE 3 Marginal zone of the spleen. (A) The strongly NSE-positive metallophilic macrophages are absent from the marginal zone of a B-cell-depleted fetal lamb. Some weak reactivity is present in a few cells. v=blood vessel. ×95. (B) The strongly NSE-positive metallophilic macrophages are prominant in the marginal zone of a control lamb. v = blood vessel. ×95. (C) Weak reactivity for ACP is present in cells of the marginal zone and red pulp of a B-cell-depleted fetal lamb. v = blood vessel. ×95. (D) Cells showing strong reactivity for ACP are present in the marginal zone and red pulp of a control lamb. v = blood vessel. ×95. (E) Strong reactivity for ALP is present in the marginal sinus of a B-cell-depleted fetal lamb. v = blood vessel. ×95. (F) Strong reactivity for ALP is present in the marginal sinus of a control lamb. v = blood vessel. ×95.

The marginal zone is an important B-cell compartment in the spleen (Kumararatne et al., 1981). The spleen of B-cell-depleted fetal lambs, which lack the strongly $IgM^+$ marginal zone B cells (Kumararatne et al., 1981; Kumararatne and MacLennan, 1981; Press et al., 1996), also lacked the strongly nonspecific esterase-positive marginal metallophilic macrophages, although some weakly nonspecific esterase-positive, acid phosphatase-positive marginal-zone macrophages did appear to be present. In mice, four subpopulations of splenic macrophages, namely, red pulp macrophages, white pulp macrophages, marginal-zone macrophages, and marginal metallophilic macrophages, have been recognized on the basis of differences in localization, membrane antigens, and repopulation kinetics after depletion (Dijkstra et al., 1985; Van Rooijen et al., 1989). The marginal metallophilic macrophages and marginal-zone macrophages have been shown to perform different roles in mounting humoral immune responses (Buiting et al., 1996) and the present findings suggest that these splenic macrophage subpopulations may also exhibit different developmental relationships to B cells. The further characterization of macrophage subpopulations in the lymphoid tissues of sheep is needed that will assist in the interpretation of changes in macrophage subpopulations in B-cell-depleted fetal sheep.

Macrophage populations in the ileal Peyer's patch also showed changes in the absence of B cells. The absence of tingible body macrophages from the follicles is easily reconciled with an absence of B cells and proliferation. However, the increased presence of macrophages in the domes of the ileal Peyer's patch is more difficult to explain. In addition to macrophages, the domes also contain many T cells, predominantly $CD8^+$ T cells and $\gamma\delta$ T cells (Press et al., 1996). An understanding of the significance of these leucocyte populations will probably depend on the further characterisation of B-cell-depleted fetal sheep and the mechanism(s) involved in the maintenance of B-cell depletion following a single treatment with an anti-IgM antibody early in gestation.

### MATERIALS AND METHODS

### Experiment Protocol

The experiment protocol has been present in detail elsewhere (Press et al., 1996). Briefly, at 63 days of gestation, the fetal sheep were exposed by caesarean section under thiopentane and halothane anaesthesia. Six fetuses each received a single intraperitoneal injection of a monoclonal antibody directed against sheep IgM (McM9) (Beh, 1988). Between 138-142 days of gestation the fetuses that had received antibody and four littermate fetuses were sacrificed by an overdose of pentobarbital via the umbilical vein. Tissue samples were collected from the ileal Peyer's patch, the spleen, the superficial cervical lymph node and the distal jejunal lymph node. The tissue specimens were frozen in isopentane (Rectapur, Prolabo, Paris), chilled in liquid nitrogen, and stored at −70°C. Sections 8 μm in thickness were cut on a cryostat and stored at −70°C until further analysis.

Four of the six antibody-treated fetal lambs showed a marked depletion of B cells in lymphoid tissues, particularly in the ileal Peyer's patch. The characterization of B-cell and T-cell populations in these fetuses has been presented in detail elsewhere (Press et al., 1996). The present study is confined to the investigation of the four B-cell-depleted fetuses and the untreated littermates. Additional control material was collected from five age-matched fetuses that had not received antibody or been subjected to surgery (gestational ages ranged from 140 to 143 days).

### Enzyme Histochemistry

Reactivities for the enzymes 5′nucleotidase, magnesium-dependent adenosine triphosphatase, acid phosphatase, alkaline phosphatase and nonspecific esterase were demonstrated using the methods given in detail in Halleraker et al. (1990).

Fifty serial sections were cut from the spleen and the superficial cervical and distal jejunal lymph nodes of the fetuses. Every tenth section was stained for 5′nucleotidase and examined for the presence of primary follicles (Nicander et al., 1991).

### Immunohistochemistry

The frozen sections were allowed to thaw and stained using an indirect peroxidase immunohistochemical method, as described in detail in Press et al. (1991). A double-immunofluorescence technique was used as described in detail in Espenes et al. (1995) with the modification that color reversal film (Kodak Ektachrome 64T EPY) was used applying double exposure with an FITC filter and a Texas red filter at the same focus. The antibodies against sheep lymphocyte determinants used in this study were a rabbit polyclonal directed against sheep IgM (#55796; Cappel Research Products, Durham, NC) and a mouse monoclonal directed against sheep complement receptor-2 (CD21, clone Du2-74; W.R. Hein, personal communication).

### *Acknowledgements*

The authors thank Elfy Gamst Bodal, Ingjerd Andersen, and Laurie Kennedy for their skilfull, technical assistance. The authors also thank W.R. Hein, Basel Institute for Immunology, Switzerland, and K. Beh, CSIRO, Australia, for providing the monoclonal antibodies used in this study.

### *References*

Beh K.J. (1988). Monoclonal antibodies against sheep immunoglobulin light chain, IgM and IgA. Vet. Immunol. Immunopathol. **18**: 19-27.

Boyd R.D.H., Haworth C., Stacey T.E. and Ward R.H.T. (1976). Permeability of the sheep placenta to unmetabolized polar nonelectrolytes. J. Physiol. **256**: 617-634.

Brambell F.W.R. (1970). The transmission of passive immunity from mother to young. In Frontiers of Biology, (Amsterdam: North-Holland), pp. 201-233.

Buiting A.M.J., De Rover Z., Kraal G. and Van Rooijen N. (1996). Humoral immune responses against particulate bacterial antigens are dependent on marginal metallophilic macrophages in the spleen. Scand. J. Immunol. **43**: 398-403.

Cerny A., Zinkernagel R.M. and Groscurth P. (1988). Development of follicular dendritic cells in lymph nodes of B cell-depleted mice. Cell Tiss. Res. **254**: 449-454.

Dijkstra C.D., Van Vliet E., Döpp E.A., Van Der Lelij A.A. and Kraal G. (1985). Marginal zone macrophages identified by a monoclonal antibody: Characterization of immuno- and enzyme-histochemical properties and functional capacities. Immunology **55**: 23-30.

Espenes A., Press C.M., Dannevig B.H. and Landsverk T. (1995). Immune-complex trapping in the splenic ellipsoids of rainbow trout (Onchorynchus mykiss). Cell Tiss. Res. **279**: 469-474.

Gerber H.A., Morris B. and Trevella W. (1986). The role of gut-associated lymphoid tissues in the generation of immunoglobulin-bearing lymphocytes in sheep. Aust. J. Exp. Biol. Med. Sci. **64**: 201-213.

Halleraker M., Landsverk T. and Nicander L. (1990). Organization of ruminant Peyer's patches as seen with enzyme histochemical markers of stromal and accessory cells. Vet. Immunol. Immunopathol. **26**: 93-104.

Halleraker M., Press C.M. and Landsverk T. (1994). Development and cell phenotypes in primary follicles of foetal sheep lymph nodes. Cell Tiss. Res. **275**: 51-62.

Kroese F.G.M., Wubbena A.S., Kuijpers K.C. and Nieuwenhuis P. (1987). The ontogeny of germinal centre forming capacity of neonatal rat spleen. Immunology **60**: 597-602.

Kumararatne D.S., Bazin H. and MacLennan I.C.M. (1981). Marginal zone: The major B cell compartment of rat spleens. Eur. J. Immunol. **11**: 858-864.

Kumararatne D.S. and MacLennan I.C.M. (1981). Cells of the marginal zone of the spleen are lymphocytes derived from recirculating precursors. Eur. J. Immunol. **11**: 865-869.

Landsverk T. (1987). The follicle-associated epithelium of the ileal Peyer's patch in ruminants is distinguished by its shedding of 50 nm particles. Immunol. Cell Biol. **65**: 251-261.

Landsverk T., Jansson Å., Nicander L. and Plöen L. (1987). Carbonic anhydrase—A marker for particles shed from the epithelium to the lymphoid follicles of the ileal Peyer's patch in goat kids and lambs. Immunol. Cell Biol. **65**: 425-429.

Landsverk T., Trevella W. and Nicander L. (1990). Transfer of carbonic anhydrase-positive particles from the follicle-associated epithelium to lymphocytes of Peyer's patches in foetal sheep and lambs. Cell Tiss. Res. **261**: 239-247.

Markgraf R., Gaudecker B.von and Müller-Hermelink H.K. (1982). The development of the human lymph node. Cell Tiss. Res. **225**: 387-413.

Nicander L., Halleraker M. and Landsverk T. (1991). Ontogeny of reticular cells in the ileal Peyer's patch of sheep and goats. Am. J. Anat. **191**: 237-249.

Nossal J.V., Abbot A., Mitchell J. and Lummus Z. (1968). Antigens in immunity. XV. Ultrastructural features of antigen capture in primary and secondary lymphoid follicles. J. Exp. Med. **127**: 277-289.

O'Neill H.C. (1994). The lineage relationship of dendritic cells with other haematopoietic cells. Scand. J. Immunol. **39**: 513-516.

Press C., McClure S. and Landsverk T. (1991). Computer-assisted morphometric analysis of absorptive and follicle-associated epithelia of Peyer's patches in sheep foetuses and lambs indicates the presence of distinct T- and B-cell components. Immunology **72**: 386-392.

Press C.M., Reynolds J.D., McClure S.J., Simpson-Morgan M.W. and Landsverk T. (1996). Fetal lambs are depleted of IgM+ cells following a single injection of an anti-IgM antibody early in gestation. Immunology **88**: 28-34.

Reid C., Stackpoole A., Meager A. and Tikerpae J. (1992). Interactions of tumour necrosis factor with granulocyte-macrophage colony-stimulating factor and other cytokines in the regulation of dendritic cell growth *in vitro* from early bipotent CD34+ progenitors in human bone marrow. J. Immunol. **149**: 2681-2688.

Reynaud C.-A., Garcia C., Hein W.R. and Weill J.-C. (1995). Hypermutation generating the sheep Ig repertoire is an antigen-independent process. Cell **80**: 115-125.

Reynaud C.-A., Mackay C.R., Müller R.G. and Weill J.-C. (1991). Somatic generation of diversity in a mammalian primary lymphoid organ: The sheep ileal Peyer's patches. Cell **64**: 995-1005.

Reynolds J.D. (1985). Evidence of differences between Peyer's patches and germinal centers. Adv. Exp. Biol. Med. **186**: 101-109.

Reynolds J.D. and Morris B. (1983). The evolution and involution of Peyer's patches in fetal and post-natal sheep. Eur. J. Immunol. **13**: 627-635.

Sakuma H., Asano S. and Kojima M. (1981). An ultrastructural study of the primary follicle in the lymph node. Acta Pathol. Jpn **31**: 473-493.

Steinman R., Adams J. and Cohn Z. (1975). Identification of a novel cell type in peripheral lymphoid organs of mice. IV. Identification and distribution in mouse spleen. J. Exp. Med. **141**: 804-811.

Van Rooijen N., Kors N., and Kraal G. (1989). Macrophage subset repopulation in the spleen: Differential kinetics after liposome-mediated elimination. J. Leuk. Biol. **45**: 97-104.

Weill J.-C., and Reynaud C.-A. (1996). Rearrangement/hypermutation/gene conversion: When, where and why? Immunol. Today **17**: 92-97.

Weiss L. (1988). Lymphatic vessels and lymph nodes. In Cell and Tissue Biology. A Textbook of Histology, Weiss L. ed. (Baltimore and Munich: Urban & Schwarzenberg), pp. 497-514.

